# Host-Induced Gene Silencing of Rice Blast Fungus *Magnaporthe oryzae* Pathogenicity Genes Mediated by the Brome Mosaic Virus

**DOI:** 10.3390/genes8100241

**Published:** 2017-09-26

**Authors:** Lin Zhu, Jian Zhu, Zhixue Liu, Zhengyi Wang, Cheng Zhou, Hong Wang

**Affiliations:** 1 Key Laboratory of Bio-organic Fertilizer Creation, Ministry of Agriculture, Anhui Science and Technology University, Bengbu 233100, China; zhulin1124@aliyun.com; 2 School of Life Sciences and Technology, Tongji University, Shanghai 200092, China; Zhujian1@mail.tongji.edu.cn (J.Z.); liuzhixue@163.com (Z.L.); 3 KWS SAAT SE, Einbeck 37574, Germany; 4 College of Agriculture and Biotechnology, Zhejiang University, Hangzhou 310058, China; zhywang@zju.edu.cn

**Keywords:** *Magnaporthe oryzae*, gene silencing, rice blast, fungal pathogens, brome mosaic virus

## Abstract

*Magnaporthe oryzae* is a devastating plant pathogen, which has a detrimental impact on rice production worldwide. Despite its agronomical importance, some newly-emerging pathotypes often overcome race-specific disease resistance rapidly. It is thus desirable to develop a novel strategy for the long-lasting resistance of rice plants to ever-changing fungal pathogens. Brome mosaic virus (BMV)-induced RNA interference (RNAi) has emerged as a useful tool to study host-resistance genes for rice blast protection. Planta-generated silencing of targeted genes inside biotrophic pathogens can be achieved by expression of *M. oryzae*-derived gene fragments in the BMV-mediated gene silencing system, a technique termed host-induced gene silencing (HIGS). In this study, the effectiveness of BMV-mediated HIGS in *M. oryzae* was examined by targeting three predicted pathogenicity genes, *MoABC1*, *MoMAC1* and *MoPMK1*. Systemic generation of fungal gene-specific small interfering RNA (siRNA) molecules induced by inoculation of BMV viral vectors inhibited disease development and reduced the transcription of targeted fungal genes after subsequent *M. oryzae* inoculation. Combined introduction of fungal gene sequences in sense and antisense orientation mediated by the BMV silencing vectors significantly enhanced the efficiency of this host-generated trans-specific RNAi, implying that these fungal genes played crucial roles in pathogenicity. Collectively, our results indicated that BMV-HIGS system was a great strategy for protecting host plants against the invasion of pathogenic fungi.

## 1. Introduction

Rice blast disease caused by the ascomycete *Magnaporthe oryzae* is a serious rice disease in more than 90 countries across the globe [1], which are devastating threats to rice production worldwide [2]. It has been estimated that each year harvest losses caused by rice blast can reach 10% to 50% of the global rice yield [3]. Over years, great progress has been gained to withstand rice blast caused by *M. oryzae* through chemical treatments. However, fungicide resistance is rapidly developed by the fungus [4]. For decades, constant efforts have been made to find rice cultivars resistant to rice blast because of the strong ability of *M. oryzae* to evolve new pathotypes. One of the most economical and sustainable routes is genetic resistance that can confront plant diseases effectively, although most genetic resistance is not everlasting in cereals. Some fungal genes play crucial roles in pathogenicity, and knockout of these genes significantly causes less virulence of pathogenic fungus to host plants [5]. A leading model has recently been established for rice blast systems to explore plant-fungus interactions due to genetic accessibility of genome sequences for both the fungus and rice [6]. Thus, there is an urgent need to develop an efficient strategy to confer more long-lasting resistance of rice to ever-changing fungal pathogens.

RNA interference (RNAi) is a conserved biological process across almost all eukaryotic organisms, in which double-stranded RNA (dsRNA) interfering molecules causes degradation of homologous mRNA molecules, and further inhibits the transcription of targeted genes [7]. It has been indicated that RNAi is an important tool for functional genomics researches in most eukaryotes such as worms, protists, animals, and plants [8,9,10,11,12,13]. Increasing evidence has indicated that the RNA silencing technology can be used in crop protection strategies [14,15]. In several insects and nematodes, RNAi reduces the expression of targeted genes by injecting dsRNA [7] and feeding with bacteria that produce the dsRNA [16,17]. For vascular plants, RNAi molecules can quickly transfer from cell to cell, and even systemically throughout the whole plant [8]. This discovery breaks through our traditional thinking that RNAs can just play a part within the cells where they are produced. Similar to other eukaryotes, most fungi are also sensitive to RNAi [18,19,20]. Intriguingly, recent studies have indicated that trafficking of RNAi molecules occurs between fungi and its host plants. The expression of endogenous genes in the infecting fungi significantly decreased in host plants carrying dsRNAs from *Blumeria graminis* or *Fusarium verticillioides* genes [21,22], indicating that the host-induced RNAi has a great promise for fighting fungal infection by expression of dsRNA of fungal genes in plants. This is thus desirable to exploit the method for control of rice blast. However, it is time consuming and laborious to generate stable RNAi plants because of long-term selection of transformants and their molecular identification. Besides, the extensive progress of *M. oryzae* genome data gives rise to the identification of candidate genes associated with pathogenesis. It has gradually been recognized that the development of effective and rapid biotechnologies is crucial to examine the functions of candidate genes responsible for disease development.

Virus-induced gene silencing (VIGS) is a suitable method to deal with these problems mentioned above. Recently, VIGS has become an important tool that triggers RNAi silencing by the use of viral vectors to generate dsRNA of targeted genes [23,24]. Because of its rapid procedure, VIGS has recently been applied to assay gene functions in plants [25]. VIGS biotechnology was primarily successful in dicots until an efficient system was developed for silencing gene expression transiently in wheat and barley mediated by the barley stripe mosaic virus (BSMV) [26]. In addition to BSMV, the brome mosaic virus (BMV) has been identified as suitable for VIGS-induced gene silencing in rice [27]. BMV is the type member of the genus *Bromovirus* with a positive-strand tripartite RNA genome constituting three RNA segments known as RNA1, RNA2, and RNA3 [28]. A new VIGS system has been established by modifying BMV vector based on the BMV to silence phytoene desaturase (*PDS*) genes in maize, barley, wheat and rice. Until now, the BMV-VIGS system is utilized to rapidly screen candidate genes in plants that determine the compatible interactions with fungal pathogens [29]. However, a VIGS system that allows efficient and systemic screening of the predicted *M. oryzae* genes vital for disease development has not been developed for rice-*M. oryzae* interaction. Nowara et al. [22] have reported that host plants expressing the dsRNAs from *B. graminis* genes exhibit a marked reduction in the transcription of targeted genes in infecting fungi. Concomitantly, the disease development is apparently suppressed by such host-induced gene silencing (HIGS). This approach is also emulated by Panwar et al. [30] to silence pathogenicity genes in the wheat leaf rust fungus *Puccinia triticina*. These studies suggest that the small interfering RNA (siRNA) molecules in planta can be transferred into fungal cells during fungal infection. Hence, the HIGS technique could be a great strategy that controls rice blast disease.

In this work, we tested the feasibility of the BMV-HIGS system to silence some putative *M. oryzae* genes vital for disease development. The rice BMV-VIGS system previously established by Ding et al. [27] was further optimized for adapting to experimental conditions, which allowed the infection with *M. oryzae*. An ATP-driven efflux pump encoded by *MoABC1* has been shown to be essential for pathogenesis for protecting itself against rice defense mechanisms [31]. *MoMAC1* encodes a membrane-bound adenylate cyclase (MAC) catalyzing the production of cAMP from ATP, which plays a central role in appressorium formation of *M. oryzae* [32]. *MoPMK1*, encoding mitogen-activated protein kinase (MAPK), is involved in the regulation of infectious hyphal growth in *M. oryzae* [33]. The three genes, *MoABC1*, *MoMAC1* and *MoPMK1*, were thus selected as candidate genes that were involved in pathogenicity. The inoculation with BMV silencing vectors remarkably repressed fungus invasion and disease development in rice plants. The HIGS efficiency significantly increased when synchronously introducing targeted gene fragments and silencing three fungal pathogenic genes together. Our results provided important evidence that the HIGS technique can be used to control rice blast disease effectively.

## 2. Materials and Methods 

### 2.1. Plant Materials and Growth Conditions

To conduct VIGS experiments, *Oryza sativa* cv. CO-39 was grown on growth chambers at 25 °C day and 22 °C night, 16 h/8 h light/dark cycles (200 μmol·m^−2^·s^–1^). Tobacco (*Nicotiana benthamiana*) seeds were sown in a soil mixture. At 3 weeks after planting, individual seedlings were transplanted into pots, and were then cultured at 25 °C with a light/dark (16 h/8 h) cycle (200 μmol·m^−2^·s^–1^). CO-39 and tobacco seeds were provided by Zhengyi Wang (College of Agriculture and Biotechnology, Zhejiang University).

### 2.2. Construction of Gene Silencing Vectors

The genome of BMV constitutes three RNA segments, including RNA1, RNA2 and RNA3. Furthermore, the constructs (pF1-11, pF2-2 and pB3m) were used to conduct BMV-mediated gene silencing as described previously by Ding et al. [27] and were provided by R. S. Nelson (Samuel Roberts Noble Foundation, Inc.). A 286-bp fragment of *OsPDS* was amplified using a pair of primers containing *Nco*I and *Avr*II restriction sites, respectively. The products of PCR amplification were digested with *Nco*I and *Avr*II, and were then inserted in sense or antisense orientation into the RNA3 component to generate pB3m-*OsPDSs* and pB3m-*OsPDSas*, respectively. For *M. oryzae* pathogenic genes, the primers were designed for *MoABC1*, *MoMAC1* and *MoPMK1*, respectively. All primers contained *Nco*I and *Avr*II restriction sites for integration into pB3m vector (Supplementary Materials Table S1). Gene fragments for inducing VIGS were amplified from the complementary DNA (cDNA) of *M. oryzae* Guy11 by PCR, and were subsequently inserted into pB3m in sense and antisense orientation, respectively. For in silico analysis of siRNA production, the SIRNA SCAN (http://bioinfo2.noble.org/RNAiScan.htm) was used. All recombinant RNA3 vectors were further confirmed by sequencing.

### 2.3. In Vitro Transcription of BMV RNAs and BMV Inoculation 

To obtain BMV RNA1, RNA2 and RNA3, three plasmids coding for BMV genome were linearized using *Spe*I (pF1-11) or *PshA*I (pF2-2, pB3m or recombinant pB3m plasmids) restriction enzymes (New England Biolabs, Beijing, China). The linearized plasmids were used to perform in vitro transcription by the mMessage mMachine T3 in vitro transcription kit (Ambion, Austin, TX, USA). Before BMV inoculation, the synthesized RNAs were verified by agarose gel electrophoresis. To achieve more successful infections, the viral transcripts were initially inoculated into *N. benthamiana* seedlings that were easily infected and allowed high titers of many viruses to accumulate in leaves. A mixture of 1 µL of each viral RNA component with 60 µL of buffer solutions (1% (*w/v*) bentonite, 1% (*w/v*) Celite, 22 mM Na_4_P_2_O_7_·10H_2_O, 60 mM K_2_HPO_4_, and 77 mM glycine) (Sangon Biotech, Shanghai, China) was inoculated into tobacco seedlings. The inoculated plants were then moved to greenhouse at 24/20 °C (day/night). After seven days of inoculation, leaf tissue from the infected *N. benthamiana* plants was separated and ground in 0.1 M phosphate buffer solution (PBS) (pH 6.5; 1:10, *w/v*) (Sangon Biotech) at 40 °C. Quantitative real time PCR (qRT-PCR) was conducted to quantify the BMV titer as reported by van der Linde et al. [29]. All crude extracts of *N. benthamiana* leaves were adjusted to the same virus titer of 10^4^ relative expression units compared with non-inoculated plants. For rice infection, leaves of 2-week-old rice seedlings were dusted with carborundum, and were then inoculated with the crude tobacco extract prepared before. The inoculated rice seedlings were placed in a greenhouse at 25/20 °C (day/night).

### 2.4. Fungal Inoculation and Phenotypic Analysis

*M. oryzae* strain Guy11 was used in our experiments and provided by Zhengyi Wang (College of Agriculture and Biotechnology, Zhejiang University). Growth conditions for *M. oryzae* were carried out, according to the method described by Talbot et al. [3]. For infection with *M. oryzae*, seedling spray inoculation and cut-leaf assays were used as reported by Wang et al. [34]. Seedling spray inoculation was conducted as follows: conidia were harvested from the plate cultures of fungus grown on complete medium (CM) medium for 12 days. 19-day-old rice seedlings (5 days after BMV inoculation) were inoculated with conidial suspensions of *M. oryzae* prepared in 0.025% Tween 20 at a concentration of 5 × 10^4^–10^5^ conidia/mL. The inoculated seedlings were then placed in a growth chamber at 14-h light periods and 25 °C with 90% relative humidity until disease symptoms occurred. Disease lesions were monitored after 10 days of fungal incubation, and 5-cm-long leaf tips were randomly selected to calculate lesion densities.

### 2.5. cDNA Synthesis and qRT-PCR

After centrifugation at 12,000× *g* for 5 min, 2 µL of leaf extracts was used for virus titer detection. The BMV titers were assayed using qRT-PCR analysis. Tobacco actin was selected as internal reference. qRT-PCR analysis was performed in a ABI 7500 fast real-time PCR system as reported recently by Zhu et al. [35]. To examine whether the recombinant RNA3 vector carrying *OsPDS* sequence triggered *OsPDS* silencing, the non-inoculated younger leaves of each inoculated rice plant were separated, and total RNA was isolated from them using Trizol reagent (Takara, Dalian, China). Further, to test the silencing efficiency of *OsPDS* in different rice leaves, total RNA was isolated from Leaf 4, 5 and 6 of each inoculated rice plant. After extraction, using the PrimeScriptTM RT Kit (Takara), about 1 µg of total RNA was reversely transcribed into cDNA as templates for qRT-PCR. *OsPDS* mRNA levels in various plants were normalized by the elongation factor 1α (*EF-1α*). In addition to assay lesion amount and the expression of targeted fungal genes, 5 cm sections of fungal infected leaves were harvested after 10 d of fungal infection (15 d after BMV inoculation). The rice *EF-1α* and β-tubulin gene (*BT*) from *M. oryzae* were selected as respective references to normalize targeted gene expression.

### 2.6. Staining and Confocal Microscopy

Leaf samples were harvested after 10 days of fungal infection (15 days post inoculation with BMV). The samples were treated with 1 M KOH (Sangon Biotech), heated to 70 °C for 20 min, and were then stained in aniline blue solution (0.05%, *w/v*) in 0.067M K_2_HPO_4_ (pH 9.0) (Sangon Biotech) for 5 min. Excessive dye was washed with sterile water. Finally, the staining samples were observed and photographed by epifluorescence microscopy. Fluorescence of aniline blue was monitored using a confocal microscope (Leica, Wetzlar, Germany) with an excitation filter of 405 nm and an emission filter of 500 nm.

### 2.7. Short Interfering RNA Detection

Small RNA molecules were examined according to the method reported by Panwar et al. [30]. Total RNA was isolated from rice plants at 15 days post inoculation with BMV. Total RNA (about 15 µg) for each sample was separated on a 15% polyacrylamide-7 M urea gel and transferred to neutral Hybond NX membrane (Amersham Pharmacia Biotech, Piscataway, NJ, USA). Probes were labelled with the phosphorus-32 deoxycytidine triphosphate ([α^32^P]-dCTP) using the DNA labelling kit (Amersham) RNA blot hybridization was performed using PerfectHyb plus Hybridization buffer (Sigma, St. Louis, MO, USA) at 42 °C overnight [36]. DynaMarker prestain (Takara) for small RNA was used as a molecular size marker. The bands were detected using a fluorescent image analyzer.

### 2.8. Statistical Analyses

Each experiment was conducted with three biological repeats. Each bar represents the mean ± standard deviation (SD), and different letters represent significant differences among different experiment groups using at a Tukey’s test at *p* < 0.05.

## 3. Results

### 3.1. Systemic Gene Silencing of PDS in Rice Mediated by BMV

**Figure 1:**
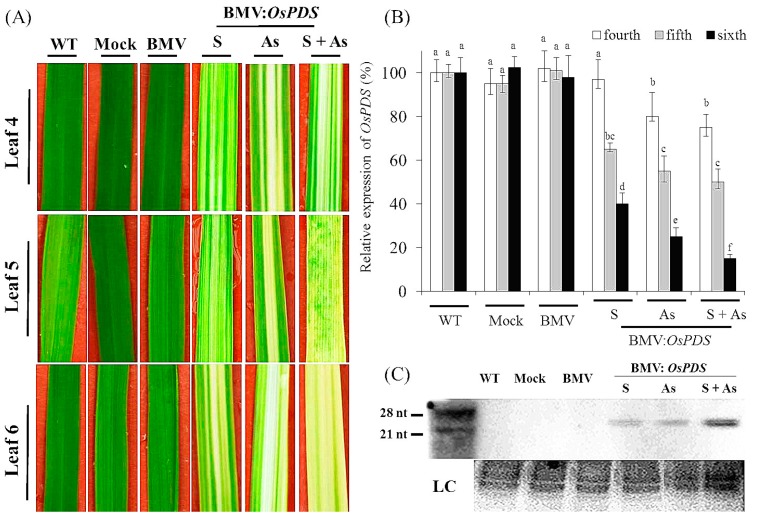
Silencing of phytoene desaturase (*PDS*) in rice CO-39 using the *Brome mosaic virus* (BMV)-induced gene silencing system. (**A**) Leaf 2 of 2-week-old rice CO-39 plants were inoculated with BMV carrying derivatives of recombinant RNA3 vector expressing gene segments of *OsPDS*s, either in antisense or as a mixture of sense plus antisense orientation. WT, Mock and BMV (without inserting) plants were considered as the controls. Photographs were taken 15 days post inoculation. *OsPDS* gene silencing caused visible photo-bleaching symptoms of upper the fourth, fifth and sixth leaf (Leaf 4, 5 and 6). Compared to the plants inoculated with BMV-*OsPDS* in sense form, more visible photo-bleaching can be seen in the plants inoculated with BMV-*OsPDS* in sense, antisense and as a mixture of sense plus antisense; (**B**) Quantitative real time-polymerase chain reaction (qRT-PCR) analyses of *OsPDS* silencing in rice leaves inoculated with BMV variants; (**C**) RNA gel blot analyses of *OsPDS* siRNA accumulation. Small RNAs were detected in rice plants inoculated with BMV variants harboring *OsPDS* insert in antisense or a mixture of sense plus antisense form. No signal was detected in WT, Mock and BMV inoculated controls. WT, wild type; S, sense; As, antisense; S + As, sense plus antisense; LC, stained rRNAs as loading controls. Each experiment was performed with three biological repeats. Values represented mean ± standard deviation (SD) in three independent experiments, and 15 individual leaves from different plants were collected for each experiment. Different letter indicated a significant difference at *p* < 0.05.

To establish BMV-based HIGS system, Ding’s rice VIGS protocol [27] was first adapted in the lab by using *M. oryzae* susceptible rice inbred line CO-39 in the experiment, and by using the *OsPDS* gene as a phenotypic marker for evaluation and optimization of the system. 2-week-old rice CO-39 seedlings at the two-leaf stage were inoculated with BMV carrying derivatives of recombinant RNA3 vector expressing target *OsPDS* gene segments. The plants treated with buffer (Mock), or treated with non-modified BMV (BMV) as well as plants not treated with BMV (wild type (WT)) were designed as controls. After 15 days of exposure to BMV infection, the plants with inoculation of BMV-*OsPDS* displayed strong photo-bleaching phenotype in newly emerged upper non-inoculated leaves (Figure 1A). More importantly, BMV infection did not appear to affect the growth of rice cultivar CO-39 (Supplementary Materials Figure S1).

To identify the best time point of *M. oryzae* infection, the efficiency of BMV-induced VIGS in different rice leaves was assessed. As shown in Figure 1A, the efficiency of VIGS seemed to depend on the tested leaves. The sixth leaf (Leaf 6) displayed the strongest photo-bleaching phenotype, and the symptoms in Leaf 6 were more apparent than in the fourth and fifth leaf (Leaf 4 and 5). To precisely quantify the silencing efficiency in different leaves, we examined the transcription levels of *OsPDS* in BMV-infected plants using qRT-PCR. Consistent with the observed phenotype, the silencing of *OsPDS* was most efficient in Leaf 6 compared with the controls; whereas this was less effective in Leaf 4 and 5 (Figure 1B). Based on the data, we defined the following infection procedure: the second leaf (Leaf 2) of 2-week-d-old rice seedlings was inoculated with BMV, and the plants were infected with *M. oryzae* for seedling spray inoculation after five days of BMV inoculation.

To further investigate whether transgene orientation affected gene silencing efficiency, the BMV carrying the sense or antisense orientation of *OsPDS* fragments (BMV-*OsPDSs* or-*OsPDSas*) was inoculated into rice plants, respectively. The *OsPDS* silencing phenotype caused by BMV-*OsPDSs* did not noticeably differ from that induced by BMV-*OsPDSas* (Figure 1A). In accordance with this, the results of qRT-PCR analyses indicated no significant difference of the *OsPDS* expression between plants inoculated with BMV-*OsPDSs* and -*OsPDSas* (Figure 1B). Furthermore, we examined the effects of the insertion of both sense and antisense *OsPDS* fragments on the silencing efficiency of *OsPDS*. For this purpose, plants were inoculated with BMV-*OsPDSs* and BMV-*OsPDSas* simultaneously. As shown in Figure 1A, the *OsPDS* silencing phenotype was even further evident in plants carrying both the sense and antisense sequence compared to the previous two treatments. qRT-PCR analyses further confirmed it: the Leaf 6 of plants inoculated with the BMV-*OsPDSs* plus BMV-*OsPDSas* constructs exhibited a reproducible and efficient silencing of average 90% compared with the controls, which was associated with the occurrence of photo-bleaching. It has previously been indicated that the generation of small RNAs is responsible for RNAi-induced gene silencing [7]. Herein, northern blotting was used to detect siRNA molecules specific to *OsPDS* in Leaf 6 of non-inoculated plants. The results showed the band of relevant siRNA production targeted, which was triggered by the inoculation of BMV (Figure 1C).

### 3.2. BMV-HIGS Allows Functional Analysis of Fungal Pathogenic Genes during the Rice-M. oryzae Interaction

**Figure 2:**
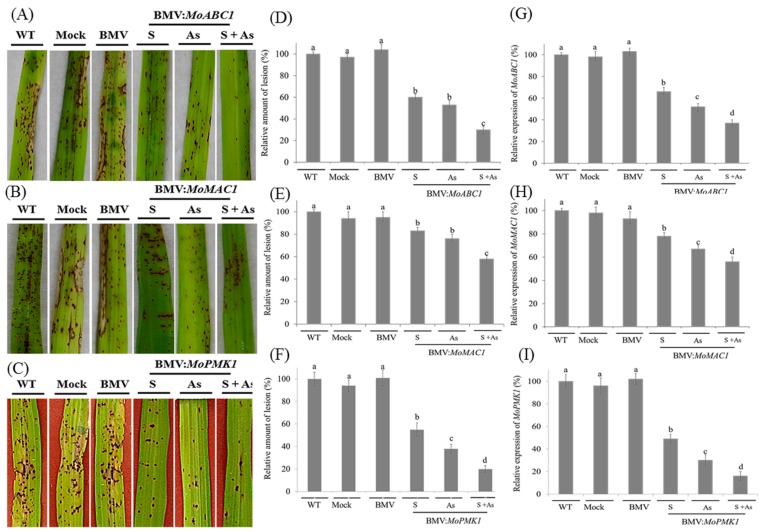
Effects of BMV-derived host-induced gene silencing (HIGS) of targeted *M. oryzae* genes on disease development in rice. Leaf 2 of 2-week-old rice CO-39 leaves were infected with BMV carrying derivatives of recombinant RNA3 construct carrying target *MoABC1*, *MoMAC1*, or *MoPMK1* gene fragments, either in sense (S), antisense (As) or as a mixture of sense plus antisense (S + As) form. WT, Mock and BMV-treated plants were selected as the control groups. After 5 days of BMV or buffer inoculation, *M. oryzae* was used to infect rice plants. (**A**–**C**) Rice blast disease phenotype in the control and silenced rice plants. Plants were treated with BMV vectors harboring the *MoABC1*, *MoMAC1*, or *MoPMK1* gene fragments. After 10 days of fungal infection, the 6th leaves had photographs taken. (**D**–**F**) Quantification of lesion density on both the BMV-infected plants carrying the targeted gene fragments and controls. After 10 days of *M. oryzae* infection, lesions in leaves from 15 independent plants were counted. (**G**–**I**) qRT-PCR analysis of the *MoABC1*, *MoMAC1*, and *MoPMK1* transcripts in rice leaves inoculated with BMV variants. Silencing was induced by BMV carrying derivatives of recombinant RNA3 vector expressing the targeted gene fragments, either in sense, antisense or as a mixture of sense plus antisense form. S, sense; As, antisense; S + As, sense plus antisense. Each experiment was performed with three biological repeats. Values represented mean ± SD in three independent experiments, and 15 individual leaves from different plants were collected for each experiment. Different letter indicated a significant difference at *p* < 0.05.

Based on the established system for BMV-induced gene silencing, several putative *M. oryzae* genes were chosen as targeted genes. Pathogen genes responsible for fungal invasions were of interest because the inhibited expression of these pathogenicity-determining genes would potentially lead to reduction of disease development. Targeting such genes would test whether the BMV-HIGS system was suitable for analyzing the functions of fungal genes. In this study, three pathogenic genes were chosen: *MoABC1*, *MoMAC1* and *MoPMK1*, respectively. Sense or antisense fragments of *MoABC1*, *MoMAC1* and *MoPMK1* coding regions were inserted into the BMV RNA3 component to generate silencing constructs. Leaf 2 of 2-week-old CO-39 plants was inoculated with BMV vectors carrying targeted gene fragments in sense or antisense orientation. By five days post inoculation, when virus symptoms were seen in upper non-inoculated leaves, seedling spray was used to perform the infection of *M. oryzae* to rice leaves, and rice blast disease symptoms were observed in the days following the inoculation (Figure 2A–C). Compared with the controls, the number of lesions was observed to distinctly decrease in rice plants inoculated with BMV vectors carrying the targeted gene fragments (Figure 2A–C). 

To examine if the efficiency of *M. oryzae* gene silencing was enhanced by introducing both sense and antisense forms of the corresponding gene fragments, rice leaves were infected with the BMV carrying derivatives of recombinant RNA3 vector expressing the pathogenic gene segments in a mixture of sense plus antisense form. In contrast to the previous two treatments, combined introduction of the sense plus antisense sequence even further suppressed rice blast symptom development. In the case of *MoABC1*, reduction of fungal disease phenotype generated by the BMV-*MoABC1as* construct did not markedly differ from that caused by the BMV-*MoABC1s* (Figure 2A), whereas the combined use of sense plus antisense forms significantly reduced the number of lesions in rice leaves compared to the previous two treatments. Compared with *MoABC1*, the reduction of fungal disease phenotype was seen less clearly in the case of *MoMAC1* (Figure 2B). The most significant reduction of fungal disease phenotype appears in the plants inoculated by the BMV carrying derivatives of recombinant RNA3 vector expressing the *MoPMK1* gene segments, especially in a mixture of sense plus antisense form (Figure 2C).

To examine the effects of *M. oryzae* gene silencing on disease phenotypes, we looked at the number of lesions on Leaf 6 of the rice plants after 10 days of fungal infection. Compared with the controls, the fungal density was remarkably decreased in rice leaves silenced for all the tested genes (Figure 2D–F). Up to statistical data, the strongest disease suppression occurred in the inoculated leaves mixed with recombinant BMV RNA3 components carrying targeted genes in sense and antisense orientation, especially in the case of *MoPMK1*. Reduction of lesion number in the rice plants inoculated with BMV-*MoPMK1s +* as construct reached above 85% compared with the controls. After 10 days of *M. oryzae* infection, the efficiency of targeted fungal genes was also detected using qRT-PCR analysis. As shown in Figure 2G–I, endogenous transcript abundance of all three corresponding *M. oryzae* genes was significantly reduced. Silencing of *MoPMK1* was the strongest when BMV-*MoPMK1s* and BMV-*MoPMK1as* were introduced simultaneously compared with introduction of the sense or antisense form alone. In the case of *MoABC1*, *MoMAC1* and *MoPMK1*, the efficiency of fungal gene silencing reached about 65%, 44% and 89%, respectively.

**Figure 3:**
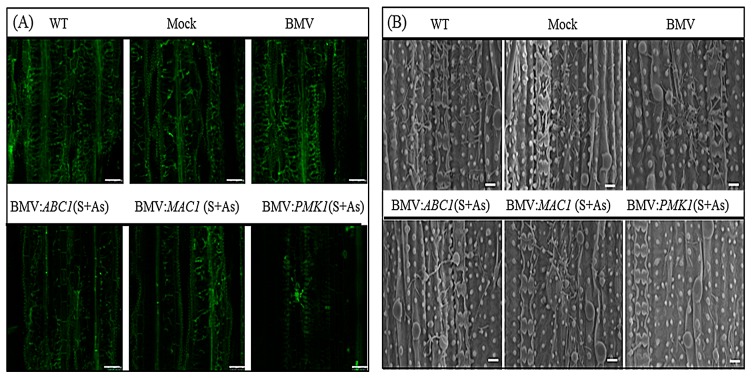
Observation of fungal development in rice CO-39 leaves. (**A**) Fungal development in rice CO-39 leaves was observed by confocal microscope. Plates represent a projection of scans taken from inside Leaf 6 of rice plants 10 days after *M. oryzae* infection but previously inoculated with BMV carrying derivatives of recombinant RNA3 vector expressing target *MoABC1*, *MoMAC1* and *MoPMK1* gene segments in as a mixture of sense plus antisense form. WT, Mock and BMV (without inserting) plants were used as controls. Silencing of the targeted *M. oryzae* genes induced by host-generated RNAi restricts fungal development whereas control plants show extensive mycelia growth. Scale bars are 10 µm. (**B**) Under a scanning electron microscope (SEM), fungal development in rice leaves inoculated with BMV variants were observed. Scale bars are 50 µm.

Reduction of leaf lesions might result from a significant inhibition of fungal growth. Thus, we further investigated the effects of BMV-induced gene silencing on the accumulation of fungi inside leaf tissue. Compared with the controls, the inoculation with BMV vectors targeting *M. oryzae* genes significantly inhibited the fungal growth and development. Considerable reduction of the lesion amount was seen in the BMV-infected leaves carrying both the sense and antisense forms of targeted sequences simultaneously. The data revealed that the growth of *M. oryzae* inside host plants was markedly impaired, which was likely attributed to the silencing of endogenous *MoABC1*, *MoMAC1* and *MoPMK1* genes. Moreover, we examined the effects of gene silencing on the fungal development in leaves; chosen sites from Leaf 6 of BMV-silenced and control plants, 10 days after fungal infection, were assayed by scanning electron microscope (SEM) and confocal microscopy. The BMV silencing vectors were used to treat rice leaves, in which *M. oryzae* infection was remarkably suppressed, indicating severe arrest of mycelial growth. In contrast, widespread fungal growth was observed in the controls. As shown in Figure 3A,B, the most significant reduction of fungal growth in the leaves appeared in the case of *MoPMK1* gene.

### 3.3. Effective Control of Rice Blast Disease by Co-Silencing of M. oryzae Pathogenic Genes

**Figure 4:**
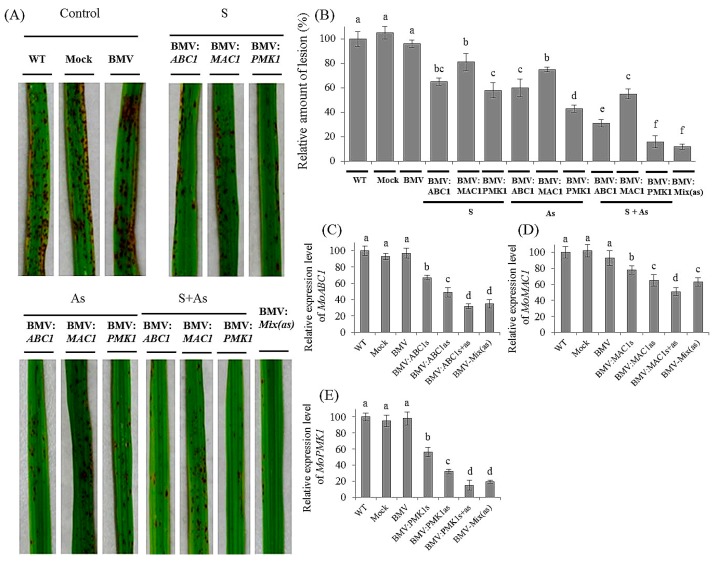
Effect of BMV-derived HIGS of targeted *MoABC1*, *MoMAC1* and *MoPMK1* on disease development in rice. Leaf 2 of 2-week-old rice CO-39 plants were infected with BMV harboring derivatives of recombinant RNA3 vector expressing *MoABC1*, *MoMAC1* and *MoPMK1* gene fragments, in sense (S), antisense (As) or as a mixture of sense plus antisense form (S + As). In addition, some plants were also inoculated with a mixture of BMV carrying derivatives of recombinant RNA3 vector expressing the targeted gene segments in antisense. WT, Mock and BMV (without inserting) plants were selected as the control groups. After 10 days of BMV infection, rice plants were treated with *M. oryzae*. (**A**) Rice blast disease phenotype in the control and silenced rice plants. Plants were treated with BMV vectors harboring candidate *M. oryzae* gene fragments. After 10 days of fungal infection, the 6th leaves had photographs taken. (**B**) Quantification of lesion density on rice plants treated with BMV vectors carrying the targeted gene fragments and controls. After 10 days of *M. oryzae* infection, lesions in leaves from 15 independent plants were counted. Furthermore, qRT-PCR analysis of the expression of *MoABC1* (**C**) *MoMAC1* (**D**) and *MoPMK1* (**E**) in rice leaves inoculated with BMV variants, including S, sense; As, antisense; S + As, sense plus antisense, and BMV-Mix(as). Each experiment was performed with three biological repeats. Values represented mean ± SD in three independent experiments, and 15 individual leaves from different plants were collected for each experiment. Different letter indicated a significant difference at *p* < 0.05.

Based on the individual gene silencing study with the BMV-HIGS system, we were curious about whether the effects of the growth inhibition of *M. oryzae* were more obvious when silencing three fungal pathogenic genes together. Rice plants were also inoculated with BMV-*MoABC1as*, BMV-*MoMAC1as* and BMV-*MoPMK1as* simultaneously. Meanwhile, we repeated all the experimental groups in the case of *MoABC1*, *MoMAC1* and *MoPMK1* genes. As shown in Figure 4A, the development of rice blast symptoms was even further reduced in the plants that had simultaneously introduced antisense forms of three targeted gene fragments compared to the previous individual experiments (Figure 4B). Silencing efficiency of targeted fungal genes was also determined by qRT-PCR analyses, 10 days after *M. oryzae* infection. In the silenced seedlings inoculated with BMV-*MoABC1as*, BMV-*MoMAC1as* and BMV-*MoPMK1as* simultaneously, a marked reduction in endogenous gene expression was observed for all three corresponding *M. oryzae* genes (Figure 4C–F).

## 4. Discussion

Chemical mutagenesis and *Agrobacterium* T-DNA or transposons insertions are the most known strategies for studying loss-of-function in fungi, which have been widely used and are also a pivotal choice for the model fungi *M. oryzae* [37,38,39]. By contrast, RNAi is a reverse genetics tool for studying functional genomics that presents many advantages and avoids many disadvantages of traditional approaches to functional analyses in *M. oryzae* [24,30,35]: (1) it is rapid; (2) it can avoid time-consuming stable fungi transformation; and (3) it can work for the studies of *M. oryzae*. In addition, it has been proved that siRNA can across the species boundary between plants to fungus and silence the expression of fungal target genes [29,30]. The knowledge about siRNA has inspired us to develop an efficient, in planta system for studying the interaction of *M. oryzae* and its rice host via use of virus vector in rice to produce designed siRNA. 

The prerequisite for such systems is a virus vector for efficient siRNA production in rice. Ding et al. [27] has developed a BMV-based system for the purpose of rice VIGS work, which is easy to operate and can systematically produce designed siRNA against the internal rice target genes. Nevertheless, it is essential to optimize Ding’s BMV system for the experimental conditions that could allow pathogenic invasion of both *M. oryzae* and BMV. In this study, 2-week-old rice plant (cultivar CO-39) at the two-leaf stage rice was used as the materials; the constant temperature and high humidity was applied to favor both BMV virus and fungal infection. Since secondary pathogen infection and pathogenic development of the fungus may be influenced by host defense [40], it is always a concern that BMV responding to host defense may intervene with the growth of host plant. It has been reported that BMV infection did not affect the susceptibility of maize plants to fungi [29,41]. Consistent with these results, we did not detect any effects on *M. oryzae* infection of BMV-inoculated plants: the defined virus titer in the experiments caused mild BMV-infected symptoms, but the symptoms of BMV-inoculated rice leaves had not been considered to influence the investigation of targeted fungal genes, depending on the assays of host *PDS* gene silencing, a practice that was implicated by Scofield and Nelson [42]. Fungal disease symptoms were observed much similar in both BMV-inoculated and control plants. These findings provided an important basis for the screening system of gene functions.

Previous studies have indicated that silencing signals stably produced from host plants can transfer into fungi from plants and inhibit the transcription of their corresponding genes in infecting fungi [22,30]. However, it is unknown if a transient expression system like BMV can produce sufficient silencing signals, and then transport into pathogens. It would dramatically facilitate the study of fungal and host plant interaction if the BMV system can possess a similar capability and be successful applied as the HIGS system. Three *M. oryzae* genes including *MoABC1*, *MoMAC1* and *MoPMK1* responsible for disease development were chosen in the study. The transcription of targeted *M. oryzae* gene fragments in rice using BMV vectors caused gene silencing of the targeted genes in the infecting fungi and blast disease suppression, indicating that the transfer of silencing signals from rice plants into *M. oryzae* cells may disturb the fungus–plant interaction. 

Interestingly, the efficiency of gene silencing was distinctly different among the three fungal genes. Our results indicated that the silencing efficiency of *MoMAC1* was greatly lower than *MoABC1* and *MoPMK1*. The possible explanation was that the extent of gene silencing detected was associated with the expression patterns of the genes. *MoPMK1* is involved in the regulation of appressorium formation and infectious hyphal growth in *M. oryzae*, and *MoABC1* plays an essential role in protecting *M. oryzae* against plant defense mechanisms after penetrating epidermal cells of host plants by infectious hyphae. However, *MoMAC1* gene is crucial for vegetative growth, conidiation, and conidial germination [42,43,44,45]. Thus, it was postulated that the silencing signal was transferred from host plant into fungi through infectious hyphae more easily, which led to the higher silencing efficiency of *MoPMK1* and *MoABC1*. A common characteristic of the fungal genes for which silencing was observed obviously was that they were transcribed highly in the infectious hyphae than that in the fungal conidia of the infecting leaves. Therefore, based on the BMV-HIGS system, selection of certain pathogenic genes transcribed highly in the infectious hyphae may be beneficial to control rice blast disease effectively. 

One of the universal concerns is whether VIGS inadvertently causes the suppression of non-target genes, so-called ‘off-target’ effects [46]. The sequences that had been selected as silencing inserts were searched against all available rice genomic database and the other pathogenic genes of *M. oryzae*, and no extensive homology was detected. In fact, no obviously aberrant phenotypes occurred in the rice plants with targeted siRNA molecules. Thus, careful selection of targeted gene sequences for the insertion of the BMV vectors may be crucial for achieving efficient and specific gene silencing. The success of pathogen gene suppression is dependent on the dynamic interplays between BMV spread and fungi infection. 

The rice seedling starts to show significant reductions in internal gene expression after 3 days of BMV inoculation and the silencing effect persists until about 21 days post inoculation [27], indicating the period of BMV mediated siRNA production in rice plants. The window for the inoculation of rice plants with *M. oryzae*, therefore, was quite wide; 15 days after BMV infection was a proper period to wait before inoculating with *M. oryzae* to achieve gene silencing. We also found that, based on the BMV-HIGS system, the silencing efficiency of targeted *M. oryzae* genes was greater with the mixture of fungal genes in sense and antisense orientation than that in sense or antisense orientation alone, indicating that such self-complementary sequences significantly enhanced the capability of dsRNA generation to increase the efficiency of BMV silencing. Thus, these data can be applied to design more effective BMV vectors for the HIGS system. 

## 5. Conclusions

Based on our data, the rice blast control was more successful when silencing more pathogenic genes in *M. oryzae* than silencing single genes. The improved understanding will be conducive to developing more effective tools against fungal infection. The results showed that some *M. oryzae* genes were markedly silenced using the HIGS system. We also proposed the BMV-HIGS as a novel strategy for studying gene function in *M. oryzae*, which owned the potential to control fungal disease. If gene silencing was confined to some particular cells, this may restrict the genes that can be assayed for their functions, and would be an increasing concern in designing constructs for engineering RNAi-triggered defenses. Taken together, the BMV-HIGS is a rapid process to promote the understanding of fungus biology and the pathogenicity of these pathogens during plant-fungus interaction.

## Supplementary Materials

The following are available online at www.mdpi.com/2073-4425/8/10/241/s1. Figure S1: Effects of BMV infection on the growth of rice CO-39 plants. 2-week-old rice CO-39 plants were inoculated with BMV vector without inserting gene fragments for 15 days. Growth phenotypes of WT, Mock and BMV (without inserting) plants were monitored (A). In addition, fresh weight (B) and length (C) of shoots and roots from these plants was examined. Each experiment was performed with three biological repeats. Values represented mean ± SD in at least three independent sample collections (*n* = 10). Different letter indicated a significant difference at *p* < 0.05. Table S1: Primers used in this study.
