# Supplementary material for: Host-Induced Gene Silencing of Rice Blast Fungus Magnaporthe oryzae Pathogenicity Genes Mediated by the Brome Mosaic Virus
Source: Genes (Basel). 2017 Sep 26;8(10):241. doi: 10.3390/genes8100241 (PMC5664091; doi:10.3390/genes8100241)
Supplement: Supplementary file 1 [file genes-08-00241-s001.pdf]

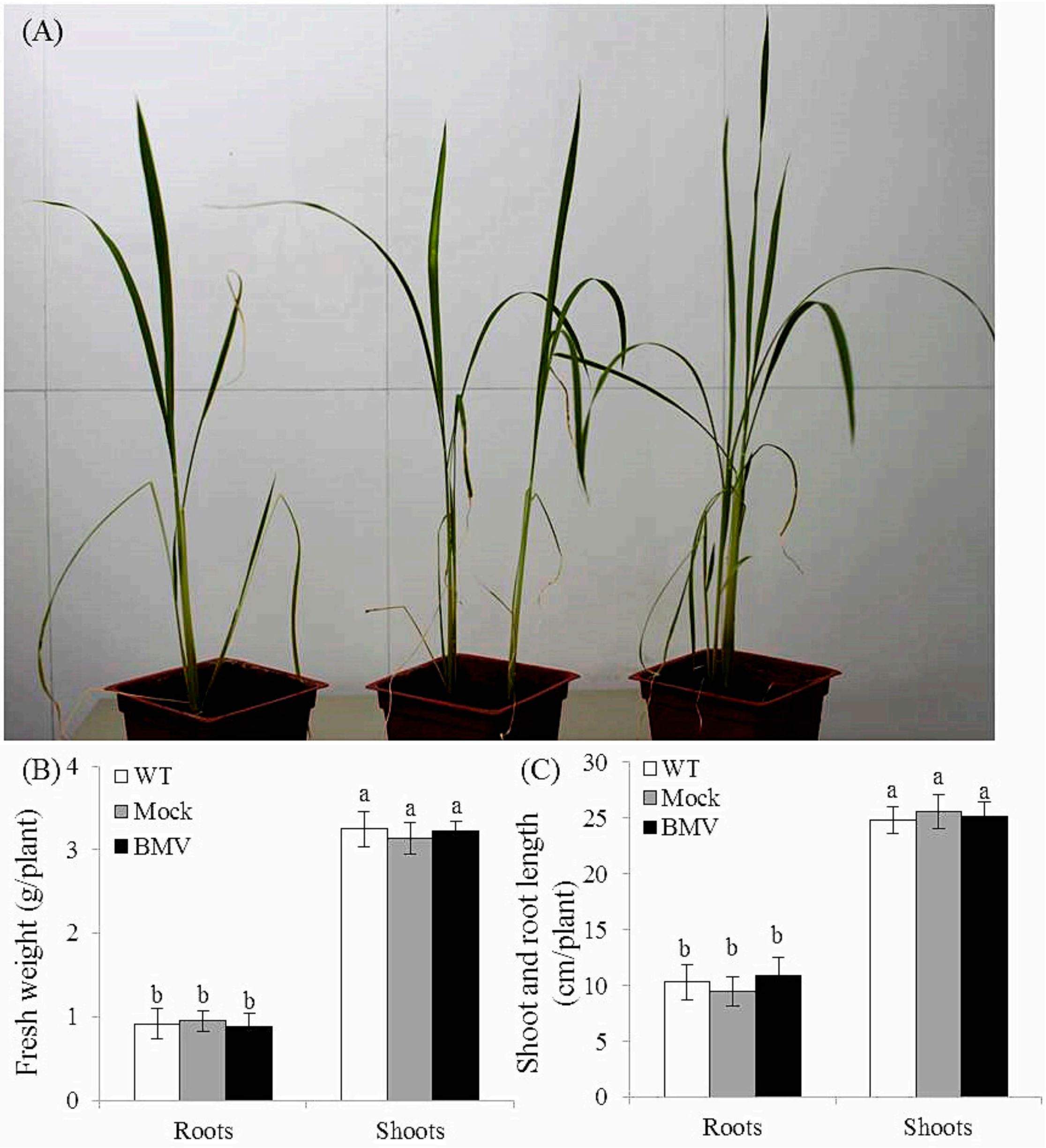

Figure S1. Effects of BMV infection on the growth of rice CO-39 plants. 2-week-old rice CO-39 plants were inoculated with BMV vector without inserting gene fragments for 15 d. Growth phenotypes of WT, Mock and BMV (without inserting) plants were monitored (**A**). In addition, fresh weight (**B**) and length (**C**) of shoots and roots from these plants was examined. Each experiment was performed with three biological repeats. Values represented mean ± SD in at least three independent sample collections (n = 10). Different letter indicated a significant difference at $p < 0.05$.

Table S1. Primers used in this study.

Gene	Usage	Sequence (5′-3′)
OsPDS(s)	Gene siliencing	CCATGGTGCCAGATATTTTGCAGGACAAC<br>CCTAGGTCCAGCAATCACGACCTGTAATG
OsPDS(as)	Gene siliencing	CCATGGTCCAGCAATCACGACCTGTAATG<br>CCTAGGTGCCAGATATTTTGCAGGACAAC
MoABC1(s)	Gene siliencing	CCATGGGGTTTCAATGGCGTGGTA<br>CCTAGGTTCGCAGCAAAGGTAAGG
MoABC1(as)	Gene siliencing	CCATGGTTCGCAGCAAAGGTAAGG<br>CCTAGGGGTTTCAATGGCGTGGTA
MoMAC1(s)	Gene siliencing	CCATGGAGGGCGAGAAGTCCACTG<br>CCTAGGTGGTGTTTGCGTAGAGCGATG
MoMAC1(as)	Gene siliencing	CCATGGTGGTGTTTGCGTAGAGCGATG<br>CCTAGGAGGGCGAGAAGTCCACTG
MoPMK1(s)	Gene siliencing	CCATGGTACTTCAACCACGAGAACATC<br>CCTAGGAGAACGAGCCAGACCAAA
MoPMK1(as)	Gene siliencing	CCATGGAGAACGAGCCAGACCAAA<br>CCTAGGTACTTCAACCACGAGAACATC
OsEF-1α	qRT-PCR	CCGCCAAGAAGAAATGAGCA<br>TCCATGCAACGAGTGCCAT
BMV	qRT-PCR	ATATGGGCTTCCAAGGTCTG<br>TCGCGGTCAAACAACATGGC
MoBT	qRT-PCR	ATTGGTGCTGCTTTCTGGCA<br>GGAAGAGCTGGCCAAAAGGA
OsPDS	qRT-PCR	CATTGCCTGCACCCTTAAAT<br>GAACCTCACCACCCAAAGAG
MoABC1	qRT-PCR	GATGCTGTCGTCGGTGTC<br>CTTGTCCGCTCTTGGTGA
MoMAC1	qRT-PCR	CCAGCGATACACGAAGACA<br>AATACATTGGAGGTGGGAGA
MoPMK1	qRT-PCR	CCAATCCACCAAGCAACT<br>TGATCTTCTTTATGGCAACC
